# The Peptide Sequence of Diacyl Lipopeptides Determines Dendritic Cell TLR2-Mediated NK Activation

**DOI:** 10.1371/journal.pone.0012550

**Published:** 2010-09-02

**Authors:** Masahiro Azuma, Ryoko Sawahata, Yuusuke Akao, Takashi Ebihara, Sayuri Yamazaki, Misako Matsumoto, Masahito Hashimoto, Koichi Fukase, Yukari Fujimoto, Tsukasa Seya

**Affiliations:** 1 Department of Microbiology and Immunology, Graduate School of Medicine, Hokkaido University, Sapporo, Japan; 2 Department of Nanostructure and Advanced Materials, Kagoshima University, Kagoshima, Japan; 3 Department of Chemistry, Graduate School of Science, Osaka University, Toyonaka, Japan; Centre de Recherche Public de la Santé, Luxembourg

## Abstract

Natural killer (NK) cells are lymphocyte effectors that are activated to control certain microbial infections and tumors. Many NK-activating and regulating receptors are involved in regulating NK cell function. In addition, activation of naïve NK cells is fundamentally triggered by cytokines or myeloid dendritic cells (mDC) in various modes. In this study, we synthesized 16 S-[2,3-bis(palmitoyl)propyl]cysteine (Pam2Cys) lipopeptides with sequences designed from lipoproteins of *Staphylococcus aureus*, and assessed their functional properties using mouse (C57BL/6) bone marrow-derived DC (BMDC) and NK cells. NK cell activation was evaluated by three criteria: IFN-γ production, up-regulation of NK activation markers and cytokines, and NK target (B16D8 cell) cytotoxicity. The diacylated lipopeptides acted as TLR2 ligands, inducing up-regulation of CD25/CD69/CD86, IL-6, and IL-12p40, which represent maturation of BMDC. Strikingly, the Pam2Cys lipopeptides induced mouse NK cell activation based on these criteria. Cell-cell contact by Pam2Cys peptide-stimulated BMDC and NK cells rather than soluble mediators released by stimulated BMDC induced activation of NK cells. For most lipopeptides, the BMDC TLR2/MyD88 pathway was responsible for driving NK activation, while some slightly induced direct activation of NK cells via the TLR2/MyD88 pathway in NK cells. The potential for NK activation was critically regulated by the peptide primary sequence. Hydrophobic or proline-containing sequences proximal to the N-terminal lipid moiety interfered with the ability of lipopeptides to induce BMDC-mediated NK activation. This mode of NK activation is distinctly different from that induced by polyI:C, which is closely associated with type I IFN-inducing pathways of BMDC. These results imply that the MyD88 pathway of BMDC governs an alternative NK-activating pathway in which the peptide sequence of TLR2-agonistic lipopeptides critically affects the potential for NK activation.

## Introduction

Natural killer (NK) cells function in early defense against various pathogens. Microbial pattern molecules activate NK cells by stimulating pattern-recognition receptors (PRRs) in NK cells or myeloid dendritic cells (mDC) [1]. mDC-mediated NK activation occurs secondary to mDC maturation, and is competent to induce NK-activating cytokines or mDC membrane molecules to facilitate reciprocal activation of mDC and NK cells [1], [2]. Toll-like receptors (TLRs) and cytoplasmic pattern sensors are PRRs that may be associated with mDC-mediated NK activation [1], [3]. In mDC, TLR3 and cytoplasmic sensors, RIG-I/MDA5 usually participate in driving NK activation in response to double-stranded (ds)RNA [4]–[6].

*Staphylococcus aureus*, a versatile Gram-positive pathogen, is reported to activate NK cells during infection [7]. *S. aureus* cell wall components including peptidoglycan, lipoproteins, and alanylated lipoteichoic acid, are inflammation inducers, and provoke the activation of host immune cells [8]. *S. aureus* cell wall pattern molecules are mainly recognized by cell-surface TLR2 and cytoplasmic nucleotide-binding oligomerization domain 2 (Nod2) in host cells, which signal the presence of bacterial infection. Mice lacking TLR2 or the adaptor protein MyD88 are highly susceptible to *S. aureus* infection [9]. The molecular basis by which *S. aureus* activates host immunity has been investigated, and lipoprotein, rather than lipoteichoic acid, is the main trigger of immune stimulation [10] that preferentially activates TLR2 in mouse cells. TLR2/MyD88 determines the pathway for activation of macrophages in mice [11]. Lipoprotein also activates TLR2 in human cells [12], [13].

The functional properties of *S. aureus* lipopeptides have been investigated in gene-disrupted mice [9], [14], [15]. TLR2, in concert with TLR1 or TLR6, is involved in their recognition [16], [17]. Two adaptor proteins, TIRAP and MyD88, deliver TLR2 signals that activate NF-κB [18], [19], which functions in cytokine induction. These studies were mainly performed in mouse macrophages, and results were essentially consistent with other biochemical studies using macrophages [20], [21]. Nonetheless, more complicated regulation may occur in other immune-related cells, including mDCs. Recent studies suggested that in mDCs, TLR2 and MyD88 are involved in NK activation that is provoked by bacterial pattern molecules [22], [23]. Our previous results also inferred that bacterial lipoproteins act as TLR2 agonists in mDC-driven NK activation [24].

In mDCs, a subset of the antigen-presenting cells, the two major arms of the innate immune signaling pathway, the MyD88 and TICAM-1 (TRIF) pathways, function in the TLR signaling [18], [19]. In addition, cytokines including IL-12, IL-15 and IFN-α/β, as well as DC-NK contact are involved in NK cell activation [25], [26]. TLR3 is a sensor of dsRNA and induces mDC maturation via TICAM-1 [4], [25]. A characteristic feature of TLR3-TICAM-1-mediated mDC maturation is liberation of IL-12, and, independent of IL-12, drives NK cell activation [4]. On the other hand, what factors participate in TLR2-MyD88-mediated mDC maturation to drive NK activation remains largely unknown.

We identified lipopeptides from Triton X-114-solubilized *S. aureus* cells [27], [28]. Since *S. aureus* lacks lipoprotein N-acyltransferase, these lipoproteins are predicted to be S-[2,3-bis(palmitoyl)propyl]cysteines (Pam2Cys) [29]. Their diacylated structure was confirmed by MS/MS spectrometry [28]. We annotated these lipoproteins by function, which largely depends on their protein sequence [30]. Based on these results, we chemically synthesized 16 Pam2Cys lipopeptides of 6–18 amino acids (a.a.) [30]. They possessed TLR2 agonistic activity, but varied in their functional potential to activate NF-κB and liberate TNF-α from human PBMC [30], yet their NK activation potential has not been determined.

This study shows that naïve NK cells are usually in a resting state, and bacterial lipoproteins trigger mDC-mediated NK activation in response to TLR2-derived cellular events. We found that mDC maturation and NK activation are strongly modulated by the amino acid (a. a.) sequence of TLR2 agonistic lipopeptides.

## Results

### Cytokines liberated from BMDC in response to Pam2 peptides

**Figure 1:**
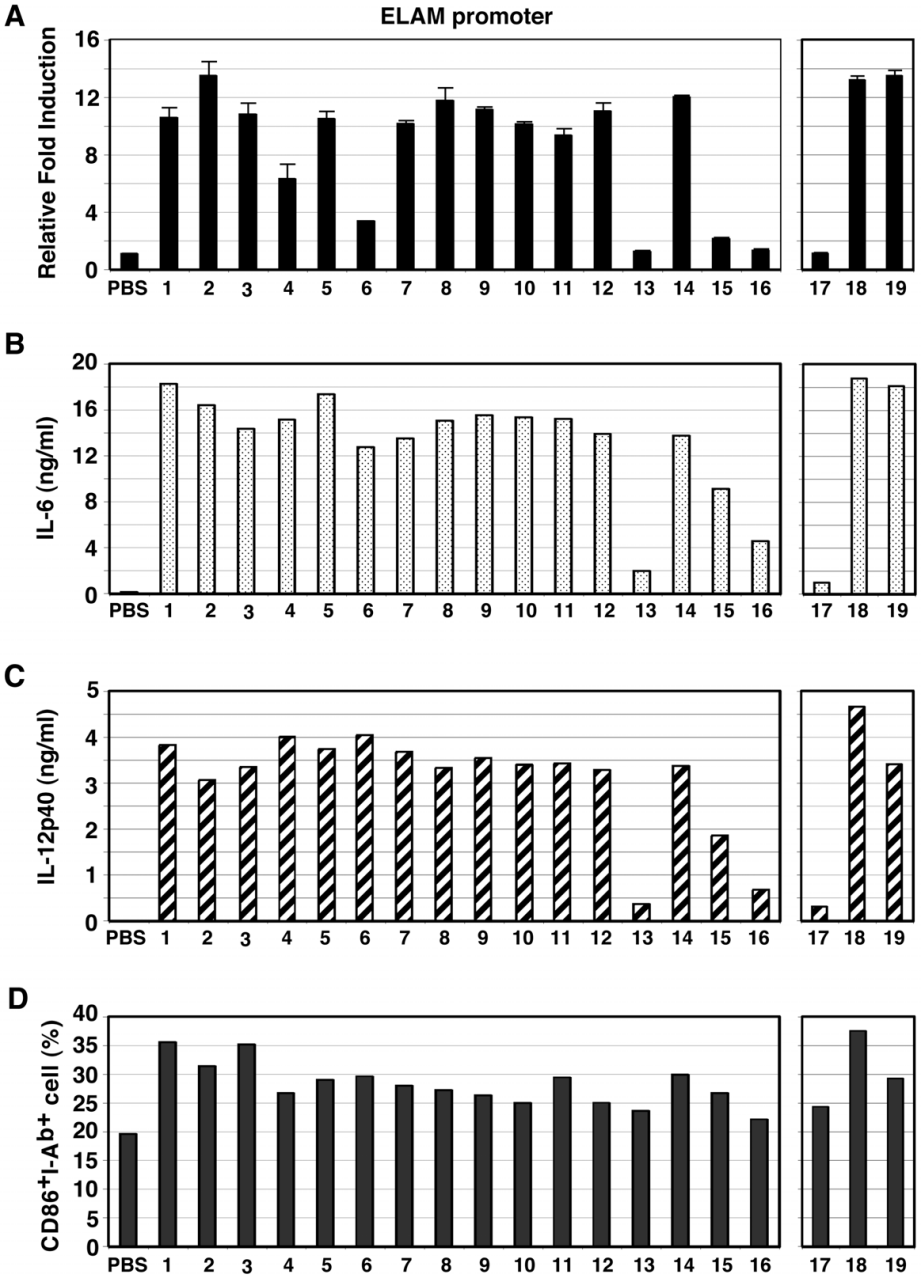
Synthetic Pam2Cys lipopeptides activate TLR2 and induce cytokine production in BMDC. (A) HEK293 cells were transfected with plasmids encoding TLR2 and ELAM-luciferase reporter. After 24 h, the cells were treated with Pam2Cys peptides (100 nM) for 6 h and then luciferase activities of the cell lysates were measured. (B, C) BMDC were treated with Pam2Cys peptides for 24 h and IL-6 and IL-12p40 concentrations in the culture supernatants were measured by ELISA. (D) CD86 and MHC class II (I-Ab) expression of the BMDC were determined by flow cytometry. Data represents the mean ± SD of triplicate measurements. The data shown are representative of at least three independent experiments. The numbers represent the Pam2Cys's numbers.

We synthesized 16 Pam2Cys-containing lipopeptides using *S. aureus* lipoproteins as a reference [30], and designated them Pam2Cys1-Pam2Cys16 (Table 1). Included as positive controls were the diacyl lipopeptides Pam2CSK4 [31], designated as Pam2CSK19 in this study, and its derivatives Pam2CSK (Pam2Cys17), and Pam2CSK2 (Pam2Cys18). Pam2Cys17 was used as a negative control, since this diacyl lipopeptide has virtually no cytokine-inducing activity (Fig. 1). ELAM-luciferase reporter assays were used to assess the NF-κB activation potential of these lipopeptides at 10–500 nM, and a representative result for 100 nM is in Figure 1A. Pam2Cys18 and 19 showed high reporter activity, but Pam2Cys17 did not (Fig. 1A). A series longer than 3 a. a were essential for TLR2 stimulation. The level of TNF-α liberated from mouse BMDC was largely comparable with that induced by human PBMC (Table 1). Pam2Cys4, Pam2Cys13, Pam2Cys15 and Pam2Cys16 exhibited relatively low NF-kB activation and TNF-α production (Table 1, Fig. 1A).

**Table 1:** Pam2 lipopeptides used for this study.

No.	Lipid	Amino acid sequence	TNF-α*
Pam2Cys1	Pam2	CANTRHSESDK	++
Pam2Cys2	Pam2	CGTGGKQSSDK	++
Pam2Cys3	Pam2	CGNGNKSGSDD	++
Pam2Cys4	Pam2	CSNIEIFNAKG	+/−
Pam2Cys5	Pam2	CTTDKKEIKAY	+++
Pam2Cys6	Pam2	CSFGGNHKLSS	++
Pam2Cys7	Pam2	CGSQNLAPLEE	+++
Pam2Cys8	Pam2	CGQDSDQQKDG	+++
Pam2Cys9	Pam2	CGNDDGKDKDG	+++
Pam2Cys10	Pam2	CGNNSSKDKEA	+++
Pam2Cys11	Pam2	CSLPGLGSKST	+++
Pam2Cys12	Pam2	CSTSEVIGEKI	++
Pam2Cys13	Pam2	CPFNCVGCYNK	+/−
Pam2Cys14	Pam2	CGSQNLAPLEEK	+/−
Pam2Cys15	Pam2	CLILIIASETL	+/−
Pam2Cys16	Pam2	CLILIIASETLFSFSHLTDVK	+/−
Pam2Cys17	Pam2	CSK	n.d.
Pam2Cys18	Pam2	CSKK	n.d.
Pam2Cys19	Pam2	CSKKKK	++

*100 pg/ml of Pam2 peptides were used for stimulation of PBMC.

TNF-α levels: +/−; <200, ++; 2,000–4,000, +++; 4,000–7,000 pg/ml.

n.d., not determined.

IL-6 and IL-12p40 levels were determined by ELISA using the supernatant of the media from bone marrow-derived DC (BMDC) culture with the lipopeptides for 24 h. The cytokines were detected at high levels in the cultures with lipopeptides, with the exception of Pam2Cys17, Pam2Cys13, and Pam2Cys16 (Fig. 1B,C). The cytokine contents of wells with BMDCs stimulated with Pam2Cys13 and Pam2Cys16 were as low as the control Pam2Cys17.

The degree of CD86 upregulation by the 16 *S. aureus* lipopeptides was examined, and similar DC maturation profiles were obtained by flow cytometer for all Pam2Cys tested (Fig. 1D), suggesting BMDC maturation, as determined by CD86 up-regulation, was independent of NF-kB activation or cytokine liberation by these lipopeptides.

### NK activation by Pam2Cys peptides

**Figure 2:**
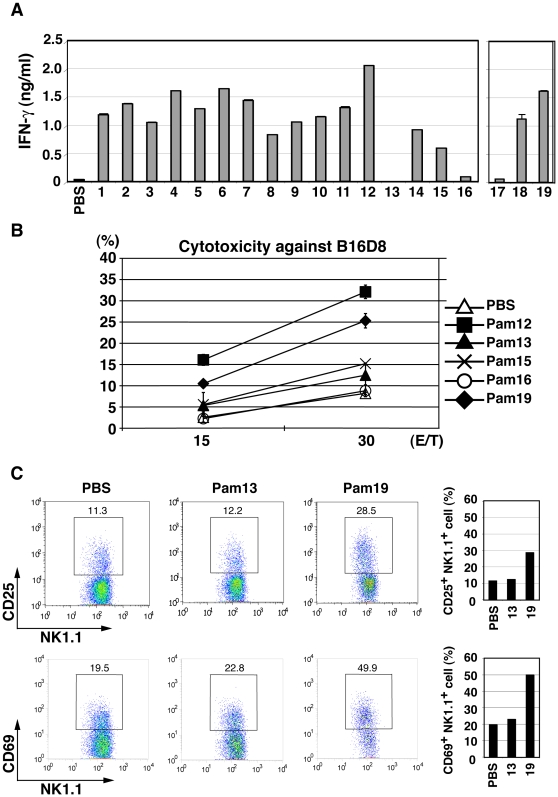
Pam2Cys peptides activate NK cells via BMDC. (A) Wild-type BMDC and wild-type NK cells were co-cultured at 1∶2 ratio in the presence of Pam2Cys peptides as the numbers indicated (100 nM). After 24 h, IFN-γ concentrations in the supernatants were measured by ELISA. (B) NK cell cytotoxicity against B16D8 cells was measured by ^51^Cr release assay at indicated E∶T ratios as described in the Methods section. (C) Populations of CD25+ and CD69+ NK cells were measured by flow cytometry after stimulation of NK cells with BMDC treated with indicated Pam2Cys peptides. BMDC were stimulated with control PBS, 100 nM of Pam2Cys13 or Pam2Cys19 for 4 h. Then, BMDC were incubated with NK cells. After 24 h, cells were analyzed by flow cytometer using the markers for separation. %Positive cells are shown to the right.

Previous reports suggested that TLR2 agonists can induce NK activation [22]–[24]. To investigate whether the *S. aureus* lipoproteins had NK cell-activating activity, we added the Pam2Cys peptides at 100 nM to BMDC/NK cultures as described previously [4]. Three markers for NK activation [26] were assessed: IFN-γ production, up-regulation of NK surface markers, and target B16D8 cell cytotoxicity by NK cells (Fig. 2). IFN-γ was generated in the supernatants (sup) in response to the lipopeptides (Fig. 2A). However, Pam2Cys13, Pam2Cys15, and Pam2Cys16 showed significantly low potential for IFN-γ induction as comparable to Pam2Cys17. Cytotoxic activity was evaluated using B16D8 cells as a target [4]. Again, Pam2Cys13, 15 and 16 did not induce effective killing (Fig. 2B). The other *S. aureus* lipopeptides had sufficient killing activity: two simultaneously generated examples are shown in Figure 2B.

The NK cell activation markers CD25 and CD69 were analyzed by flow cytometry after co-culturing NK cells with BMDC and Pam2Cys stimulants (Fig. 2C). Up-regulation of surface CD25 and CD69 was observed in NK cells incubated with BMDC and Pam2Cys18 or 19, while the levels of their up-regulation by Pam2Cys13, 15 or 16 were near those of the negative control Pam2Cys17, for stimulating NK cells co-cultured with BMDC. In contrast, no increase was observed for CD56, NKp46 and DNAM-1 (data not shown).

### Participation of TLR2/MyD88 in Pam2Cys-mediated BMDC and NK activation

**Figure 3:**
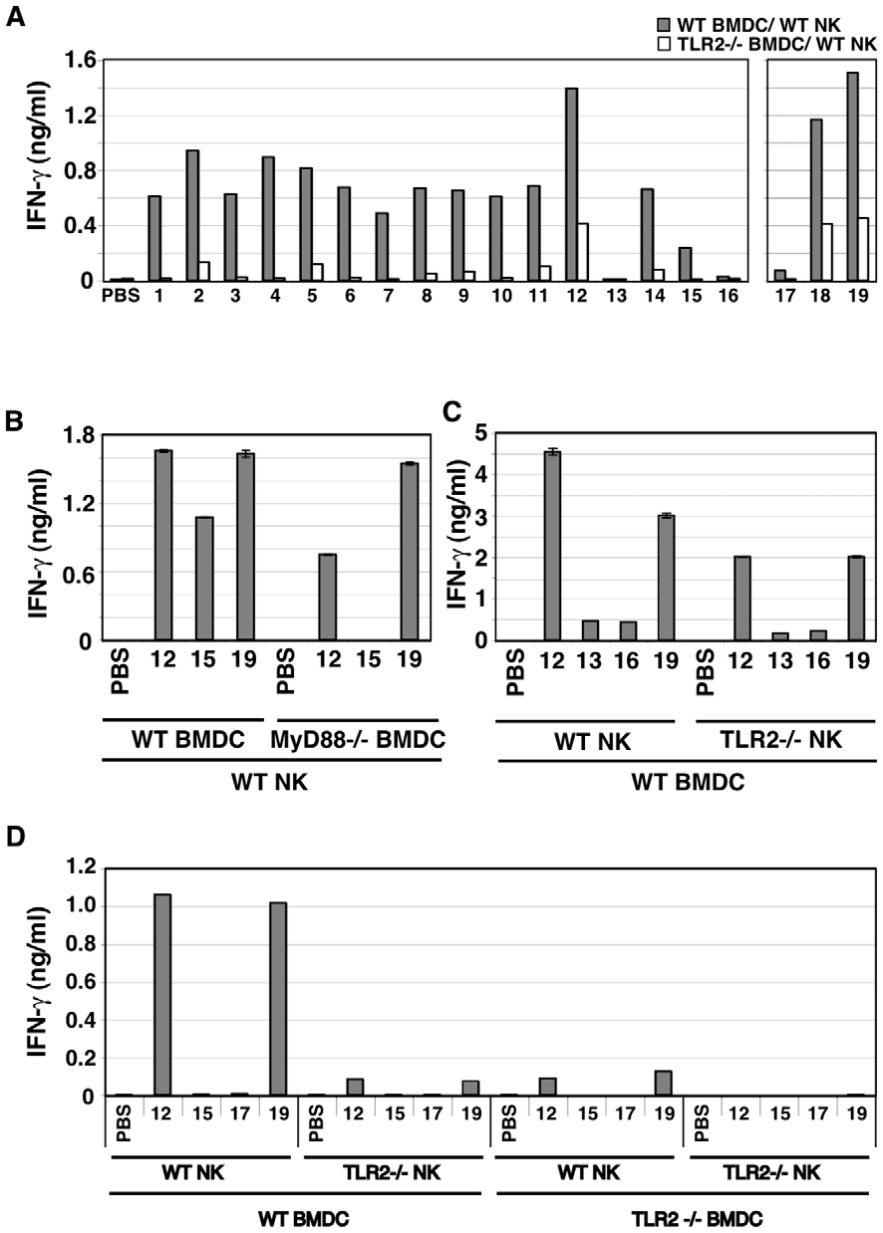
TLR2 on BMDC mainly participate in Pam2Cys-mediated NK activation. (A) BMDC TLR2-independent NK activation by Pam2Cys12, 18 and 19. BMDC from wild-type (closed bars) or TLR2−/− (open bars) mice were stimulated with control PBS or 100 nM of indicated Pam2Cys peptides for 4 h. Cells were then co-cultured with wild-type NK cells at 1∶2 ratio for 24 h. Then, the supernatants were collected and IFN-γ was measured by ELISA. (B) Pam2Cys12 and 19 induce NK activation in culture with MyD88−/− BMDC. NK cells were co-cultured with wild-type or MyD88−/− BMDC in the presence of the indicated Pam2Cys peptides (represented by the numbers) as in Fig. 2. 24 h after incubation, culture media were collected to determine cytokines by ELISA. (C) Pam2Cys12 and 19 induce TLR2−/− NK activation in culture with wild-type BMDC. Wild-type BMDC and NK cells with either wild-type or TLR2−/− phenotype were incubated at 1∶2 ratio with the indicated Pam2Cys peptides (represented by the numbers) as in Fig. 2. 24 h after incubation, culture media were collected to determine cytokines by ELISA. One representative of the three similar experiments is shown. (D) TLR2 NK cells mainly participates in TLR2 BMDC-independent NK activation by Pam2Cys12 and 19. Wild-type and TLR2−/− BMDC were stimulated with indicated Pam2Cys peptides for 4 h. These BMDC were then mixed with NK cells as shown in the panel. Four groups consisting of either of wild-type NK or TLR2−/− NK and either of wild-type BMDC or TLR2−/− BMDC (see the bottom of the panel) were incubated with the indicated Pam2Cys peptides (50 nM) for 24 h. NK cells alone with Pam2Cys12 or 19 liberated minure IFN-γ as in the panel with WT NK + TLR2−/− BMDC (see Fig. S1). IFN-γ concentrations in the culture supernatants were determined by ELISA. The data shown are representative of at least three independent experiments.

Activated NK cells are a major source of IFN-γ, which causes a variety of responses in the immune system. To examine whether direct stimulation of NK cells with Pam2Cys18 or Pam2Cys19 induced secretion of IFN-γ, we measured the frequency of IFN-γ-secreting NK cells, at 24 h after incubation. By intracellular staining, IFN-γ-secreting NK cells were increased after direct Pam2Cys18 or 19 stimulation (data not shown). As shown in Figure 3A and B, TLR2 ligands except Pam2Cys12, 18 and 19 barely increased the levels of IFN-γ of NK cells by co-culture with Pam2Cys-stimulated TLR2−/− or MyD88−/− BMDC. On the other hand, NK cells induce moderate levels of IFN-γ in response to BMDC stimulated with Pam2Cys12, 18 or 19 (open bars in Figure 3A), although no structural similarity was detected between Pam2Cys12 and Pam2Cys18 or 19.

We next examined whether lipopeptide-mediated cytokine secretion and NK activation were dependent on BMDC TLR2 and MyD88. IL-6 and IL-12p40 secretion were completely abrogated in TLR2−/− BMDC (data not shown). However, low amounts of IFN-γ were detected in co-cultures of TLR2−/− or MyD88−/− BMDC and wild-type (WT) NK cells in the presence of Pam2Cys12, 18, or 19 (Fig. 3B,D), and lesser extent of IFN-γ was still detected in co-cultures of WT BMDC and TLR2−/− NK cells in the presence of Pam2Cys12 or Pam2Cys19 (Fig. 3C). These results were reproduced with MyD88−/− BMDC (not shown). Notable results are shown in Fig. 3D where WT or TLR2−/− BMDC were stimulated with indicated Pam2Cys and incubated with WT or TLR2−/− NK cells. Moderate IFN-γ was detected in the media containing TLR2−/− BMDC, WT NK and Pam2Cys12 or 19 (Fig. 3D), the IFN-γ levels being comparable to those of WT NK cells alone stimulated with Pam2Cys12 or 19 (Fig. S1). TLR2−/− NK cells still produced very low levels of IFN-γ when the TLR2−/− NK cells were co-cultured with WT BMDC (Fig. 3D). However, No IFN-γ was detected in the media containing TLR2−/− NK and Pam2Cys12 or 19. Thus, all Pam2Cys peptides including Pam2Cys12, 18 and 19 act on BMDC to drive NK activation. Notably, WT NK cells alone produce minute IFN-γ in response to Pam2Cys12 or 19 (Fig. S1), which means that Pam2Cys12 and Pam2Cys19 additionally induce direct NK activation. The Pam2Cys receptors for this NK activation is through the NK cell TLR2 followed by the MyD88 pathway. However, minute activation by Pam2Cys12 or 18/19 appears to be left in TLR2−/− NK cells stimulated with Pam2Cys12/19-treated BMDC, which should be attributed not to TLR2 but to other unknown mechanisms.

### Combinatorial recognition of Pam2Cys lipopeptide by TLR2 and TLR6

**Figure 4:**
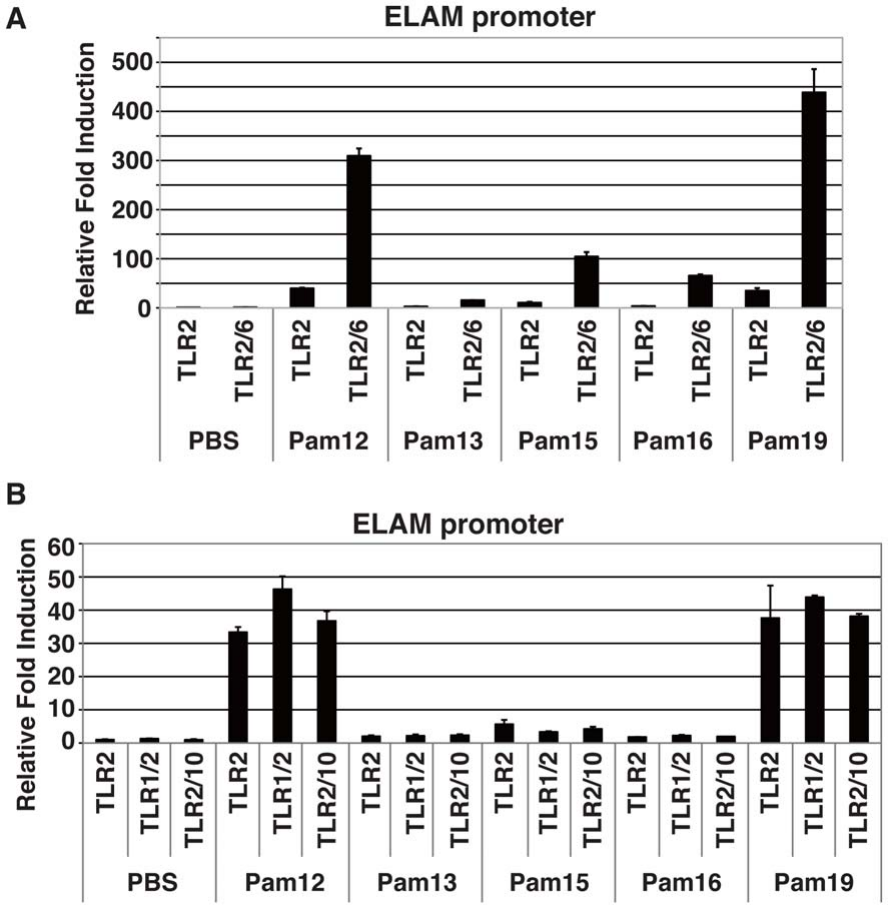
TLR6 promotes TLR2 signaling in Pam2Cys peptides recognition. (A, B) HEK293 cells were transfected with plasmids encoding TLR2, TLR6, TLR1 or TLR10 and the ELAM-luciferase reporter. After 24 h, the cells were treated with indicated Pam2Cys peptides (100 nM) for 6 h and then luciferase activities were measured. The data shown are representative of at least three independent experiments.

TLR2 recognizes diacyl lipopeptides in combination with TLR6 [14], [31] while TLR2 recognizes triacyl lipopeptide with TLR1 [15], [32]. We found TLR2/6 cooperation in the recognition of *S. aureus* lipopeptides using HEK293 cells that express TLR2/6. Data on the use of TLR2/6 by Pam2Cys12, Pam2Cys13, Pam2Cys15, Pam2Cys16, and Pam2Cys19 is shown in Figure 4. Single receptors of TLR1, TLR6, and TLR10 showed little activation of NF-κB by reporter assay, and only TLR2 exhibited <60-fold ELAM promoter activation (data not shown). Pam2Cys12 and Pam2Cys17 more efficiently activated the ELAM promoter (>300 fold over the control) with TLR2 and 6, than with TLR2 alone (Fig. 4A). TLR1 or TLR10 in combination with TLR2 did not amplify the signal (Fig. 4B). Pam2Cys13 only weakly enhanced the TLR2 potential with additional TLR6 expression in HEK cells (Fig. 4A), and only a slight increase was observed with Pam2Cys15 and Pam2Cys16 with simultaneous expression of TLR2 and TLR6 (Fig. 4A). Hence, TLR6 helped TLR2 to amplify the TLR2 signal from most Pam2Cys lipopeptides, but not with Pam2Cys13 or Pam2Cys15/16.

### Critical a.a. in Pam2Cys lipopeptides for BMDC-mediated NK activation

**Figure 5:**
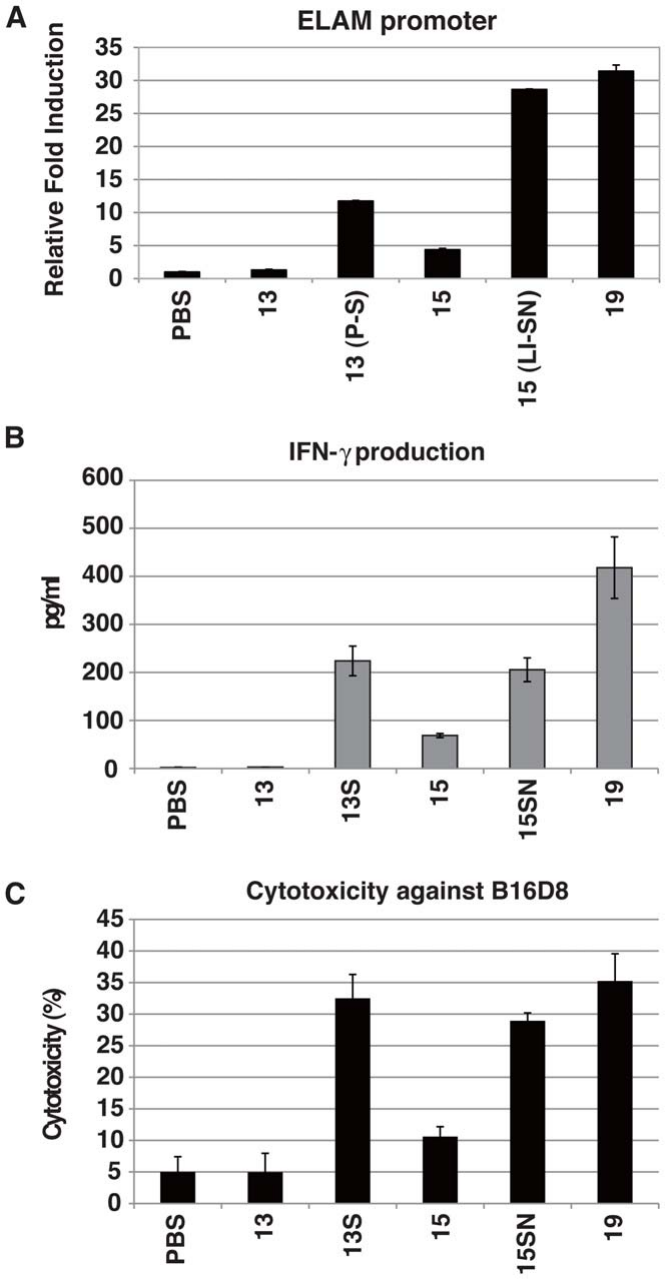
Amino acids near the Pam2 lipid are critical for TLR2 recognition. (A) HEK293 cells were transfected with plasmids encoding TLR2 and the ELAM-luciferase reporter. After 24 h, the cells were treated with indicated Pam2Cys peptides (100 nM) for 8 h and then luciferase activities were measured. The numbers represent the Pam2Cys's numbers. 13(P-S), Pam2Cys13 with second Proline replaced with Serine; 15(LI-SN), Pam2Cys15 with second Leucine and third Isoleucine replaced with Serine and Asparagine. (B,C) BMDC-mediated NK cell activation occurs by stimulation with Pam2Cys13 (P-S) and Pam2Cys15 (LI-SN). BMDC and NK cells were prepared from wild-type mice. BMDC were stimulated with PBS or indicated Pam2Cys peptides for 4 h. Then, BMDC were incubated with wild-type NK cells for 24 h. IFN-γ production (A) and B16D8 cytotoxicity (E∶T ratio = 50∶1) (B) were measured as in Figure 2. 13S and 15SN represent Pam2Cys13(P-S) and Pam2Cys15(LI-SN), respectively.

Recent studies revealed an extensive cross-talk between NK cells and mDCs [2], [6]. We analyzed the structural background that supports NK activation using our synthetic diacyl lipopeptides. All NK-activating lipopeptides tested had Cys-Ser/Thr or Cys-Gly/Ala in their N-terminus (Table 1). However, the two lipopeptides with the lowest ability to activate NK cells had differences, with Cys-Pro in the N-terminus of Pam2Cys13, and Cys-Leu-Ile in Pam2Cys15/16. When the second Pro in Pam2Cys13 was replaced with Ser, and the Leu-Ile sequence of Pam2Cys16 was replaced with Ser-Asn, the newly synthesized peptides, Pam2Cys13(P-S) and Pam2Cys16(LI-SN), recovered their ELAM reporter activity (Fig. 5A).

We next tested whether BMDC mature to activate NK cells through BMDC's TLR2 activation by these modified Pam2Cys. Pam2Cys13(P-S) and Pam2Cys15(LI-SN) recovered NK-activating properties by the amino acid conversions judged by IFN-γ production (Fig. 5B) and cytotoxicity against B16D8 cells (Fig. 5C). Since Pam2Cys13(P-S) acts only on BMDC (not shown), this Pam2Cys activity is attributable to recovered BMDC maturation. Hence, Pam2Cys13(P-S) and Pam2Cys16(LI-SN) are NK activators via mDC TLR2. Hence, we conclude that the peptide sequence near the N-terminus is important for NK activation by diacyl lipopeptide.

**Figure 6:**
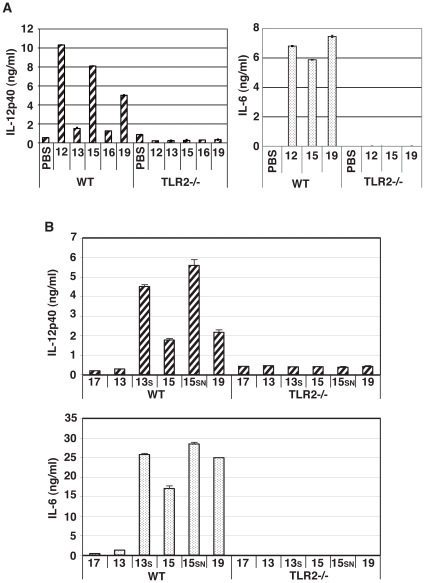
TLR2 agonists in BMDC is crucial for IL-6 and IL-12 production. (A) IL-6 and IL-12p40 production by wild-type but not TLR2−/− BMDC by Pam2Cys stimulation. BMDC prepared from wild-type or TLR2−/− mice were treated with indicated Pam2Cys peptides (100 nM) for 24 h. IL-12p40 and IL-6 concentrations in the supernatants were determined by ELISA. (B) Pam2Cys13(P-S) but not Pam2Cys13 induces IL-6 and IL-12 from BMDC. BMDC prepared from wild-type mice were treated with indicated Pam2Cys peptides (100 nM) for 24 h as in panel A. Cytokines in the supernatants were determined by ELISA. 13S, Pam2Cys13(P-S); 15SN, Pam2Cys15(LI-SN).

Production of both IL-6 and IL-12p40 was dependent on BMDC TLR2 (Fig. 6A). Pam2Cys13 and Pam2Cys16 induced these cytokines at very low levels. When Pam2Cys13(P-S) or Pam2Cys16(LI-SN) replaced Pam2Cys13 or Pam2Cys16 in the same assay system, the cytokine levels recovered to levels similar to those of the other lipopeptides (Fig. 6B). These activities were almost completely abrogated in TLR2−/− BMDCs. Thus, the a.a. replacements allows BMDC to generate the TLR2 signal, irrespective of their artificial modifications.

### BMDC-NK contact is indispensable for BMDC TLR2-mediated NK activation

**Figure 7:**
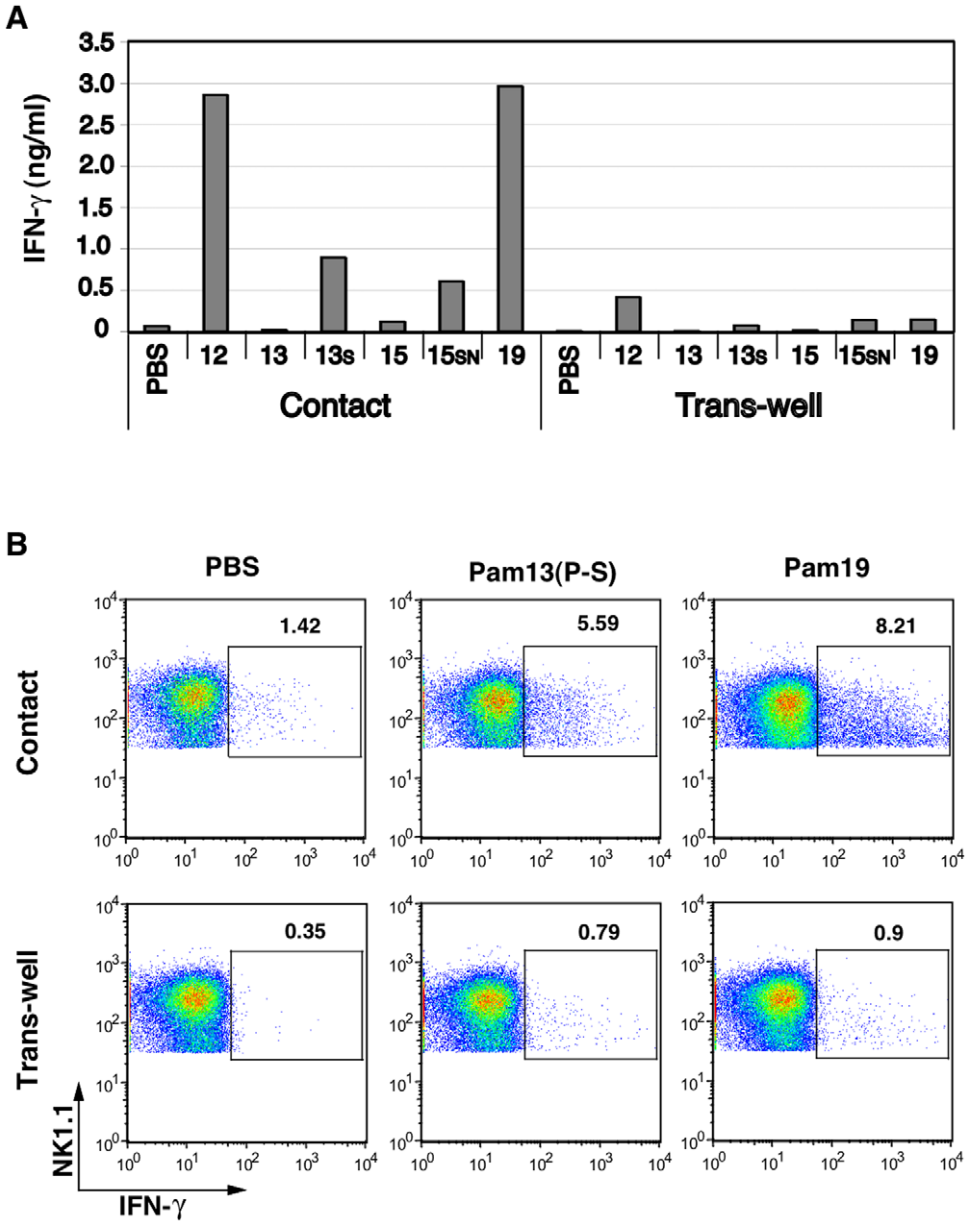
BMDC-NK cell contact induces NK activation. (A) IFN-γ induction by NK cells requires BMDC-NK contact. BMDC prepared from wild-type mice were treated with indicated Pam2Cys peptides (100 nM) for 4 h. Then, BMDC were incubated with naïve NK cells for 24 h at the ratio of 1∶2 (left hand bars in Contact). The level of IFN-γ was measured by ELISA. Of note, the sup of the stimulated BMDC was collected and added to cultures of unstimulated BMDC and naïve NK cells, but only a minute level of IFN-γ was detected in the sup (data not shown). The levels of IFN-γ in the same combinations are shown (right hand bars in Trans-well) when stimulated BMDC and NK cells were separated by trans-well. 13S, Pam2Cys13(P-S); 15SN, Pam2Cys15(LI-SN). (B) %IFN-γ-positive NK cells were determined by intracellular staining. Wild-type BMDC prepared from C57BL/6 mice were treated with Pam2Cys13(P-S) peptides (100 nM) for 4 h as in panel A. Then, wild-type NK cells were added to the wells. 20 h after co-culture, breferdin was added to the wells and incubation was continued further for 4 h. NK cell activation was determined by IFN-γ produced in NK cells. %IFN-γ-positive NK cells was determined by FACS.

Pam2Cys13(P-S) matured BMDC to activate NK cells without direct action on NK cells (Fig. 7A). First, we collected the sup of BMDC stimulated with Pam2Cys13 or Pam2Cys13(P-S). Surprisingly, Both of the sup failed to confer NK activating function on the mixture of naïve BMDC and NK cells (data not shown). The capacity of BMDC sup to induce IFN-γ-secretion by NK cells was further evaluated using a transwell system (Fig. 7A,B). No significant increase in IFN-γ-secreting NK cells was observed when lipopeptide-activated BMDCs and NK cells were separated by transwell (Fig. 7A,B). Frequency of IFN-γ-producing NK cells was high in co-culture with Pam2Cys13(P-S)-stimulated BMDC and NK cells (Fig. 7B upper panel) while the IFN-γ-producing NK cells were diminished in the transwell (Fig. 7B lower panel). In either case, IL-15 and IFN-α/β were barely increased in Pam2Cys-stimulated BMDCs by RT-PCR (data not shown). Thus, soluble factors barely participate in BMDC-mediated NK activation. BMDC-NK contact is essential for TLR2-mediated NK activation.

## Discussion

Here, we demonstrated that the a. a. sequence of *S. aureus* Pam2Cys peptides critically affects the agonistic function for TLR2 and the mode of NK activation. This NK activation is largely dependent on TLR2/MyD88 in BMDC in the mouse system. In addition, Pam2Cys12 and Pam2Cys18/19 have a weak ability to directly activate NK cells without participation of BMDC. In contrast, we determined Pam2Cys13, Pam2Cys15, and Pam2Cys16 to be dysfunctional, since these lipopeptides failed to activate TLR2/6 reporter signaling or induce cytokines in BMDC (Fig. 1,4). Although the first Cys is conserved in all the lipopeptides tested, the following sequences varied, even though all showed BMDC maturation activity. Notably, the second a. a. residue was Ser/Thr or Gly/Ala in the functional lipopeptides, followed by undefined sequences. A length of more than three a.a. was indispensable for BMDC-mediated NK activation (Fig. 1, Pam2Cys17 vs. 18). The failure of Pam2Cys13, Pam2Cys15, and Pam2Cys16 to activate NK cells suggests the importance of the second and/or third residue for stimulating BMDC or directing NK activation. Pam2Cys13 harbors Pro in the second residue, which breaks hydrogen bond interactions. Likewise, Pam2Cys15 and Pam2Cys16 commonly possess Leu and ILe in the second and third residues, which also destabilize hydrogen bond interactions. Thus, these a. a. residues critically influence the effectual interaction between the Pam2Cys peptides and the TLR2 complex on either BMDC or NK cells.

TLR2 initiates immune response by recognizing diacylated lipoproteins in combination with TLR6. We surmise that this receptor complex recognizes the a. a. properties in the peptide sequence that activate mDC/NK cells. Crystal structure analysis indicates that hydrogen bonds between glycerol and the peptide backbone of the ligand and the leucine-rich repeat (LRR)11 loops of TLRs are critical for TLR heterodimerization [31], [32]. These hydrogen bonds bridge TLR2 and TLR6 with the ligand, and fix the conformation of the hydrophobic residues around the dimerization interface [31]. The side chains of the first two a. a. of Pam2Cys have substantial interactions with the TLRs. The N-terminal Cys binds to the sulfur site formed by the hydrophobic F325, L328, F349, L350, and P352 residues of TLR2, and the L318 residue of TLR6 [31]. The hydroxyl side chains of the second Ser/Thr form a medium-range hydrogen bond with the F325 backbone of TLR2. As seen in the TLR2/TLR6/Pam2CSK4 structure, the side chains beyond the third lysine residue have highly flexible structures and form only weak ionic or hydrogen bond interactions with the TLRs [31]. Hence, our results with a. a. substitutions fit the proposed TLR2-Pam2CSK4 interacting model.

In a previous report, lysines in the triacyl peptide were seen to form hydrogen bonds with TLRs when Pam3CSK4 interacts with TLR2/TLR1 heterodimer [32]. The ε-amino residues in the side chains appear to participate in lipopeptide recognition by TLRs [31], [32]. In our results, the small side chains of Asn or Gly had no blocking effects on the peptide-TLR2 interaction (data not shown). Thus, a hydrophilic or small a. a. in the chain barely altered the lipopeptide function exerted through TLR2. The *S. aureus* lipopeptides, with the exception of Pam2Cys13, Pam2Cys15, and Pam2Cys16 are compatible with this principle. We actually demonstrated here that the peptide sequences have a significant effect on the immunological activity of the lipopeptides. The recognition system for bacterial lipoproteins has developed to sense common structures of the lipid, as well as the peptide sequences.

The factor that determines whether mDC TLR2 or NK TLR2 predominates in diacyl lipopeptide activation has remained to be settled. The sequence information about the NK-activating vs. DC-activating lipopeptides in this study suggests that lysines or hydrophilic a. a distal to Pam2Cys are involved in this selective activity. Peptides with lysines tended to exert direct NK activation. According to the structural studies [31], [32], the lysines in Pam3CSK4 form multiple hydrogen bonds with TLR2/1, and the same is expected to be true of Pam2CSK4 and TLR2/6. Hence, hydrophilic residues after the second residue of the Pam2Cys lipopeptides may participate in high affinity interaction with NK cell TLR2/6.

In a previous report, we identified the NK-activating protein INAM, which discrminates between active and resting NK cells, and reflects *in vivo* tumoricidal action of NK cells [33]. INAM is expressed on polyI:C-matured mDCs in response to IRF-3 activation, and is not expressed on mDCs stimulated with TLR2 agonists [33]. In both TLR2- and TLR3-stimulation, IFN-γ is a marker of NK activation [4], [6], [22], [33], almost parallel to NK cell-mediated target cytotoxicity. According to these criteria, mDC drive NK activation via two arrays, MyD88 and TICAM-1 pathways. Anyhow, the MyD88-mediated NK activation occurs independently of TICAM-1-mediated NK activation. Here we provide further knowledge on the MyD88-mediated NK activation: mDC TLR2-dependent, and mDC TLR2-independent pathways exist for NK activation. The latter involves NK TLR2-dependent NK activation, where the lipopeptides directly stimulate TLR2 and the MyD88 pathway in NK cells. However, a minimal NK-activating capacity by Pam2Cys12, 18 and 19 retains in TLR2−/− NK cells. This means that the direct NK activation largely depends on NK cell TLR2/MyD88 but does not neglect participation of other undetermined factors, such as NOD-like receptor (NLR) family, either in mDC or NK cells.

Pam2Cys-matured BMDC produce IL-12 by the MyD88 pathway. Unexpectedly, however, results from transwell studies did not support the importance of IL-12 in NK activation. In addition, NK-activating cytokines, such as IL-15 and IFN-α/β, are barely increased in Pam2Cys-stimulated BMDC (data not shown). Cell-cell contact rather than soluble mediators is crucial for mDC TLR2-mediated NK activation in this study.

Taken together, we showed that *S. aureus* lipopeptides induce mDC-mediated NK activation. It is intriguing that this is a case of the reported reciprocal activation [2], in which ligands-receptors on mDC and NK cells are involved. In a. a. sequences of Pam2Cys, lysine distal to the N-terminal Pam2 and hydroxyl residues proximal to the Pam2 affect NK-activating potential through its interaction with TLR2. When bacteria invade host tissue, they encounter many proteases. Since plasma serine proteases frequently cleave the Lys-X sequence of substrates, the lipoproteins may be clipped out into liberated lipopeptides containing Lys, which could be important in the context of TLR2-induced inflammatory immune responses. In fact, after completing this manuscript, two in press papers were released where some bacterial components are shown to participate in TLR2-mediated NK activation [34], [35]. Our findings furthered these notions by analyzing synthetic Pam2Cys peptides under the knowledge of the structural background of TLR2 [31], [32].

A question remaining is why bacteria provide two sorts of lipopeptides with TLR2-activating and –nonactivating properties. So far, we have no experimental finding to sufficiently answer this question, but bacterial infection usually alters host inflammatory milieu and recruits immune competent cells to the lesion [36]. In this context, it is not surprising that Pam2Cys lipopeptides serve as modifiers for host immune response against bacteria. Different responses could be expected to occur with various combinations of Pam2Cys peptides in infectious lesion. Here we demonstrate that NK activation is a phenotype induced by TLR2-activating bacterial lipopeptides, which properties are determined by the peptide sequence of the Pam2Cys. Studies on these functional behaviors of lipopeptide towards mDC and on how TLR signals link NK activation in bacterial infectious diseases will be the next highlight for understanding the importance of early phase of innate cellular response against various bacterial infections.

## Materials and Methods

### Reagents and antibodies

The following materials were obtained as indicated: Fetal calf serum (FCS) from Bio Whittaker (Walkersville, MD), mouse granulocyte-macrophage colony-stimulating factor (GM-CSF) from PeproTech EC, Ltd (London, UK), polymyxin B from SIGMA-Aldrich (St. Louis, MO), Pam2CSK4 was in part purchased from Amersham Pharmacia Biotech (Piscataway, NJ) and Lympholyte-M from Cedarlane (Ontario, Canada). The enzyme-linked immunosorbent assay (ELISA) kits for mouse (m)IFN-γ from eBioscience (San Diego, CA), and IL-12p40 and IL-6 from Amersham Biosciences.

The following antibodies were used: mAbs against mouse CD11c, NK1.1, CD86, I-Ab, CD25, CD69, DNAM1, CD56 and NKp46 were purchased from BioLegend (San Diego, CA).

### Preparation of synthetic peptides

The synthesis of lipopeptides was achieved with a combination of solution- and solid-phase methods [30]. Briefly, for the preparation of the Pam2Cys backbone, the protected cysteine (Fmoc-Cys-OtBu) and the iodide (3-iodopropane-1,2-diol) were coupled under basic condition by using Cs_2_CO_3_ to give Fmoc-Cys(2,3-dihydroxypropyl)-OtBu, and the subsequent acylation and cleavage of the tBu group gave Fmoc-Pam2Cys-OH [37]. The peptide component, which included 16 different peptide sequences from 14 lipoproteins of *S. aureus* NCTC8325, was prepared by using solid-phase synthesis on Wang resin in a similar fashion to Jung's lipopeptide synthesis. Fmoc-Pam2Cys-OH [30], [37] was then introduced to the N terminus of the peptides linked to the resin. Subsequent cleavage of the Fmoc group, detachment from the resin, and deprotection of all protecting groups gave the lipopeptides Pam2Cys1–16, and also Pam2CSK4. Pam2CSK and Pam2CSK2 were obtained from Biologica Co. Ltd. Commercial and our synthetic Pam2CSK4 had indistinguishable potential for BMDC maturation and cytokine production (data not shown).

### Mouse and cell lines

TLR2 −/−, TLR4 −/−, and MyD88 −/− mice were gifts from Dr. S. Akira (Osaka Univ., Osaka) as previously reported [9]. TICAM-1 (TRIF) −/− and IPS-1 −/− mice were established in our laboratory [4], [33]. Female C57BL/6 mice were purchased from Clea Japan (Tokyo). Mice were maintained in our institute under specific pathogen-free conditions. All animal work was performed under guidelines established by the Hokkaido University Animal Care and Use Committee. This study was approved as Analysis of immune modulation by toll-like receptors by this committee. Mice (12 weeks female C57BL/6) were housed four per cage and allowed food and water ad libitum. The Ethics committee in Hokkaido University approved this study (ID number: 08-0243). Animal studies were carefully performed without ethical problems.

B16D8 were established in our laboratory as a subline of the B16 melanoma cell line [38]. This subline was characterized by its low MHC levels with no metastatic properties when injected s.c. into syngeneic C57BL/6 mice [4], [33]. HEK293 and B16D8 cell lines were obtained from ATCC (USA). These cell lines were cultured in RPMI 1640/10% FCS.

### Preparation of BMDCs and spleen NK cells of mice

Mouse bone marrow-derived DC (BMDC) were prepared as described previously [4]. Spleen NK cells were positively isolated from spleens with DX5 Micro Beads (Miltenyi Biotech) [33]. In experiments requiring high purity NK cells, Thy1.2 beads were additionally used for negative selection according to the Miltenyi's protocol. The purity of NK cells (DX5^+^ cells) was routinely about 70%. NKT cells might be an only trace constitution of our preparation. DX5^+^ NK cells were used within 24 h.

### Reporter assay

Plasmids (pEFBos) for expression of human TLR1, TLR2, TLR6 and TLR10 were prepared in our laboratory as described previously [39]. HEK293 cells were seeded onto 24-well plates and transfected with 0.1 µg TLR expression vectors, 0.1 µg of ELAM-1, and 0.05 µg of phRL-TK control plasmid using FuGene HD (Roche) according to the manufacturer's instructions. The ELAM-luciferase reporter plasmid was made in our laboratory [13]. After 24 h, the cells were harvested in 50 µl lysis buffer. The luciferase activity was measured using Dual-Luciferase Reporter assay systems (Promega) and was shown as the means ± S.D. of at least three experiments.

### Statistical analysis

Student's *t* test was used to examine the significance of the data when applicable in quantitative studies. Differences were considered to be statistically significant when *P*<0.05.

### ELISA, Flow cytometric (FACS) analysis of cell surface antigens

The levels of cytokines (IL-6, IL-12p40, IFN-γ etc.) were determined by sandwich ELISA (Amersham Pharmacia Biotech, Buckinghamshire, UK) or the message levels assessed by quantitative PCR [38]. Surface CD25, CD86 and CD69 levels were determined by FACS using specific mAbs. The practical methods for FACS were described previously [40]. Intracellular staining of IFN-γ was peformed with breferdin-treated NK cells as described previously [39]. Trans-well analysis was performed as described previously [40]. Assays were usually performed at least three times in duplicate, otherwise indicated in the legends, and one representative experiment is shown.

### Assessment of in vitro cytolytic activity

The cytolytic activity of spleen NK cells was determined by ^51^Cr assay as described previously [4], [33]. NK cells were prepared from the spleen of intact C57BL/6 mice. NK cells were co-cultured with BMDCs at a ratio of 2∶1 and 24 h later the mixtures were subdivided to assess NK-mediated cytotoxicity [4]. A B16 subline (D8) or YAC-1 was used as a target cell. Target cells (2×10^3^ cells/well) were coincubated with NK cells at the indicated lymphocyte to target (E/T) cell ratio (typically 5, 15 and 30) in U-bottom 96-well plates in a total volume of 200 µl of 0.5% BSA/RPMI-1640 medium at 37°C. Four hours later, the liberated ^51^Cr in the medium was measured using the scintillation counter. Specific cytotoxic activity was obtained by the formula: Specific cytotoxic activity (%)  = [(experimental ^51^Cr activity - spontaneous ^51^Cr activity)/(total ^51^Cr activity – spontaneous ^51^Cr activity)] ×100. Each experiment was done in triplicate to confirm reproducibility of the results, and representative results are shown. *t* test was used to examine the significance of the data.

## Supporting Information

Figure S1 Direct activation of NK cells by stimulation with Pam2Cys12, 18 or 19. Wild-type or TLR2−/− NK cells (5×105 cells/well) were stimulated with indicated Pam2Cys peptides for 24 h. After 24 h, IFN-γ concentrations in the supernatants were measured by ELISA as in Fig. 2A. The IFN-γ concentrations were more than 5-fold lower than those in the mixture of BMDC and NK cells (see Figs. 2A and 3).
(0.08 MB TIF)
