# Supplementary figures and images for: The Peptide Sequence of Diacyl Lipopeptides Determines Dendritic Cell TLR2-Mediated NK Activation
Source: PLoS One. 2010 Sep 2;5(9):e12550. doi: 10.1371/journal.pone.0012550 (PMC2932735; doi:10.1371/journal.pone.0012550)

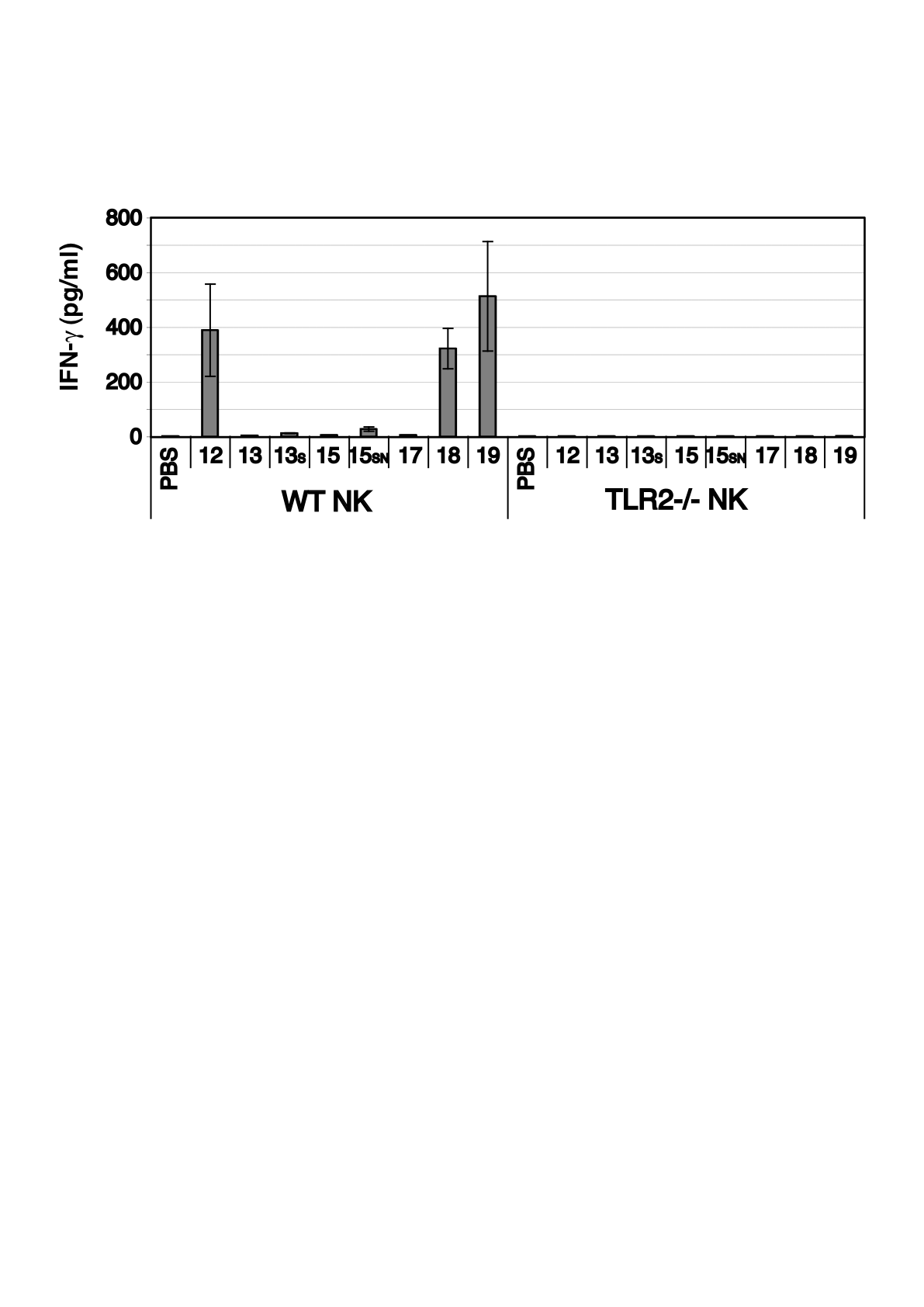

Supplement: Figure S1 — Direct activation of NK cells by stimulation with Pam2Cys12, 18 or 19. Wild-type or TLR2−/− NK cells (5×10^5^ cells/well) were stimulated with indicated Pam2Cys peptides for 24 h. After 24 h, IFN-γ concentrations in the supernatants were measured by ELISA as in Fig. 2A. The IFN-γ concentrations were more than 5-fold lower than those in the mixture of BMDC and NK cells (see Figs. 2A and 3). (0.08 MB TIF) [file pone.0012550.s001.tif]
